# Assessment of selected muscle damage markers and zonulin concentration after maximum-intensity exercise in men with type 1 diabetes treated with a personal insulin pump

**DOI:** 10.1007/s00592-023-02157-1

**Published:** 2023-07-22

**Authors:** Bartłomiej Matejko, Łukasz Tota, Małgorzata Morawska-Tota, Tomasz Pałka, Maciej T. Malecki, Tomasz Klupa

**Affiliations:** 1 Department of Metabolic Diseases, Jagiellonian University Medical College, ul. Jakubowskiego 2, 30-688 Krakow, Poland; 2 University Hospital in Krakow, Krakow, Poland; 3 Department of Physiology and Biochemistry, University of Physical Education in Krakow, Krakow, Poland; 4 Department of Sports Medicine and Human Nutrition, University of Physical Education in Krakow, Krakow, Poland

**Keywords:** Type 1 diabetes, Maximum-intensity exercise, Muscle damage, Zonulin, Personal insulin pump

## Abstract

**Aim:**

Exercise-induced muscle damage depends on exercise intensity and duration and on individual susceptibility. Mechanical and metabolic stress may disturb the intestinal microflora. The study evaluated selected muscle damage markers and zonulin concentration after maximum-intensity exercise in type 1 diabetes (T1D) men compared with healthy controls.

**Methods:**

The study involved 16 T1D participants and 28 controls matched by age (22.7 [21.3–25.1] vs. 22.6 [20.9–26.3] years), body mass index (24.2 ± 1.6 vs. 24.2 ± 1.9 kg/m^2^), and body fat percentage (16.1 ± 5.2 vs. 14.9 ± 4.6%). The T1D group had 11.3 ± 5.1 years of diabetes duration and a suboptimal mean glycated haemoglobin level of 7.2 ± 1.1%. The subjects underwent a graded running treadmill test until exhaustion. Lactate concentration was assessed in arterialized blood at baseline and 3 and 20 min after the test. Cortisol, testosterone, tumour necrosis factor α, myoglobin, lactate dehydrogenase, zonulin, and vitamin D levels were evaluated in cubital fossa vein blood before and 60 min after the test.

**Results:**

T1D patients presented higher baseline zonulin, myoglobin concentration, testosterone/cortisol ratio, and lower maximal oxygen uptake. On adjusting for the baseline values, the groups differed in zonulin, lactate dehydrogenase, and myoglobin levels, testosterone/cortisol ratio, and lactate concentration determined 20 min after exercise (*P* < 0.05).

**Conclusion:**

Maximum-intensity exercise increased muscle and intestinal damage in T1D participants. In patients with lower physical activity, very-high-intensity exercise should be recommended with caution. Observing the anabolic-catabolic index may help individualize effort intensity in T1D individuals.

## Introduction

People who are physically active-whether they are amateurs or professionals-are exposed to various ailments of the digestive, muscular, and respiratory systems [1, 2]. The energy crisis, as well as dehydration and hyperthermia are phenomena that increase mechanical and metabolic stress during physical activity. The hypothesis of mechanical stress that occurs during physical activity indicates that exercise-induced muscle damage (EIMD) results from physical stress on muscle fibres, while the metabolic stress model presents EIMD as a consequence of metabolic deficiencies [3]. Cell structure impairment leads to muscle fibre damage and pro-inflammatory cytokine release [3]. EIMD magnitude, time course, and impact on performance are variable and depend on the intensity and duration of the exercise and on the individual’s susceptibility to the damaging stimulus [4]. Mechanical and metabolic stress, as well as a reduction in gastrointestinal blood flow in favour of increased activity of other organs may cause disturbances in the intestinal microflora [5]. Side effects affecting the stomach and intestines occur in many athletes and may make them avoid exercise [6].

Type 1 diabetes mellitus (T1D) is a chronic progressive disease that results in increased mortality [7]. Among the primary efforts to achieve metabolic control in T1D, the International Society for Pediatric and Adolescent Diabetes and the American Diabetes Association recommend taking at least 60 min of moderate to vigorous aerobic physical activity daily [8, 9]. Physical activity helps alleviate the increased risk of cardiovascular complications related to T1D [10]. The cardiovascular benefits provided by physical exercise are somewhat limited in insulin-treated patients, as exercise management can be challenging owing to the risk of hypoglycaemia. However, from the clinical point of view, it seems crucial to understand the mechanical and metabolic response to exercise of varying intensity in a T1D patient [11]. In the literature, there are few studies that describe muscle cell damage induced by exercise in T1D [12]. Increased damage of muscle cells, expressed by elevated biomarker levels, can often be a challenge in the context of maintaining normal blood glucose concentrations [12]. People with T1D, compared with non-diabetic individuals, exhibit different gut microbiota, which is associated with intestinal permeability changes, inflammation, and insulin resistance [13]. Therefore, it is essential to understand the influence of physical activity on the composition and structure of the gut microbiota in patients with T1D.

Furthermore, T1D patients present a greater increase in lactate and cortisol levels after high-intensity intermittent exercise compared with moderate-intensity exercise [14]. Interestingly, EIMD can lead to transient insulin resistance (caused by inflammatory factors) also in healthy subjects [3].

The pleiotropic nature of vitamin D suggests the presence of vitamin D receptors in almost all human cell nuclei [15]. Calcitriol is responsible for a number of important functions in of the body, including bone mineralization, hormone production, and proper functioning of the nervous, immune, endocrine, cardiovascular, and muscular systems [16]. In the literature, there are many inconsistent scientific reports suggesting a relationship between plasma vitamin D concentration and the level of markers reflecting damage to muscle cells after exercise [17–19]. Moreover, these studies were conducted in healthy populations, and there are no scientific reports concerning T1D individuals.

The purpose of this study was to evaluate markers of damage to the intestines and muscles after a single session of exercise at a maximum intensity among men with T1D compared with healthy males.

## Methods

### Participants

The study involved 16 men with T1D (consecutive patients receiving medical care in the authors’ clinic were invited to participate) and 28 controls (all being university students from Krakow) matched by age and body composition. All individuals with T1D were treated with a personal insulin pump (there is a dedicated inpatient clinic at the authors’ centre that focuses on modern technologies, which is why most T1D patients receive pump treatment). The maximal aerobic capacity (VO_2_max) in the T1D group was determined as described in earlier publications [20, 21]. It should be noted that 2 h prior to the test, the patients consumed a specified meal which consisted of 50 g of carbohydrates: 30 g of cereal yogurt (255 kcal) and 20 g of bananas (116 kcal). The patients had not been trained in this form of exercise. The same exercise test was applied to the control group. The participants underwent a graded running test on a mechanical treadmill until subjective feeling of maximum exhaustion. Muscle damage markers and zonulin concentration were assessed before and after the graded test.

The inclusion criteria for the study involved male sex, the presence of T1D, treatment with a personal insulin pump for at least 1 year, the latest glycated haemoglobin (HbA1c) level of < 75 mmol/mol (9%), a lack of advanced chronic complications, and signed written informed consent. The assessment of chronic diabetes complications was based on a combination of medical records, previous tests, and consultations conducted at different points of time. No further examinations were performed specifically for this study, apart from the initial eligibility visit. The exclusion criteria for this study were as follows: pre-proliferative or proliferative retinopathy, autonomic neuropathy, polyneuropathy, stage 3 or higher chronic kidney disease, severe hypoglycaemia or diabetic ketoacidosis within 7 days prior to the testing, significant locomotor disorders, body mass index of ≥ 35 kg/m^2^, resting electrocardiogram abnormalities, as well as ineligibility for examination by an internal medicine physician. The patients’ glucose levels were continuously monitored with the use of a blinded Dexcom G4, starting from 10 days before the maximum-intensity exercise test until the end of the exercise.

### Laboratory tests

The material for biochemical tests was blood collected from a cubital fossa vein (7 ml) by a laboratory diagnostician in accordance with the applicable standards. Blood was collected to Vacutainer EDTA tubes before the maximum-intensity exercise test and 60 min after its completion. It was allowed to clot at room temperature and then centrifuged. The resulting serum was aliquoted and frozen at − 80 °C for later analyses. Then, the levels of inflammation and muscle damage markers (cortisol, testosterone, tumour necrosis factor α [TNF-α], myoglobin, lactate dehydrogenase [LDH], vitamin D) and zonulin concentration were determined. All indicators were evaluated with the enzyme-linked immunosorbent assay (ELISA) by using a DRG microplate reader (E-Liza Mat 3000, Medical Instruments GmbH, Germany).

Lactate concentration was assessed in arterialized blood collected from a fingertip before the exercise test and 3 and 20 min after its completion. The concentration of lactate in plasma was determined with the enzymatic method by using a Lactate PAP kit (BioMérieux, France) and a Spekol 11 spectrophotometer (Carl Zeiss Jena, Germany).

### Statistical analysis

To compare 2 independent variables, Student’s or Welch’s *t*-test was performed for normally distributed (Shapiro–Wilk test) continuous variables; otherwise, the Mann–Whitney *U*-test was applied. To test for the estimated difference in outcome between the groups, an ANCOVA was conducted, with adjustment for the group and baseline values. Once significant interactions were confirmed, they were included into a model, and the adjusted mean between-group difference was estimated.

The correlations between the biochemical parameters analysed were verified with the Pearson or Spearman correlation. The study results are presented as arithmetic means ± standard deviations. The results were considered significant at the significance level of *P* < 0.05. The analyses were performed with the R software, version 4.1.0, and R-Studio, version 1.3.959.

## Results

The studied groups were matched by age, body mass index, and percentage of body fat. The T1D group presented diabetes duration of 11.3 ± 5.1 years and a suboptimal metabolic control: mean HbA1c level of 7.2 ± 1.1% and mean glucose concentration with continuous monitoring from 10 days before the test of 155 ± 69 mg/dl (with mean sleep duration of 7.4 h).

At baseline, the T1D patients were characterized by a higher zonulin and myoglobin level and testosterone/cortisol ratio, and lower VO_2_max. Differences between the groups are shown in Table 1.

**Table 1:** Comparison of the T1D and matched control groups with respect to general characteristics, as well as muscle damage markers and zonulin concentration

Variable	T1D group (*n* = 16)	Control group (*n* = 28)	*p*-value
Age (years)	22.7 (21.3–25.1)	22.6 (20.9–26.3)	0.779
BMI (kg/m^2^)	24.2 ± 1.6	24.2 ± 1.9	0.971^a^
Body fat (%)	16.1 ± 5.2	14.9 ± 4.6	0.448^a^
VO_2_max/kg of body mass (l/min/kg)	45.0 (40.9–46.1)	49.5 (47.4–54.3)	< 0.001
Lactate, baseline (mmol/l)	1.6 ± 0.2	1.6 ± 0.2	0.959^a^
Lactate, 3 min post-exercise (mmol/l)	13.7 ± 3.4	12.9 ± 2.9	0.406^a^
Lactate, 20 min post-exercise (mmol/l)	7.6 (5.2–10.5)	5.8 (5.0–7.3)	0.062
Vitamin D (ng/ml)	29.3 ± 18.0	32.4 ± 12.6	0.511^a^
Zonulin, baseline (ng/ml)	12.4 (10.3–15.0)	10.0 (9–11.2)	0.015
Zonulin, post-exercise (ng/ml)	12.5 (11.5–16.7)	13.8 (9.8–19.0)	0.800
Δ Zonulin (ng/ml)	1.4 (0.5–1.9)	2.3 (0.7–8.6)	0.111
LDH, baseline (U/l)	169.1 (155.0–206.8)	156.0 (136.4–182.3)	0.116
LDH, post-exercise (U/l)	250.5 (216.1–300.5)	171.0 (151.6–205.4)	< 0.001
Δ LDH (U/l)	86.7 (55.0–108.9)	21.5 (3.9–45.0)	< 0.001
TNF-α, baseline (pg/ml)	2.94 (2.22–4.02)	2.81 (1.84–3.75)	0.538
TNF-α, post-exercise (pg/ml)	4.77 (3.77–7.44)	5.16 (2.98–6.96)	0.461
Δ TNF-α (pg/ml)	2.5 (1.3–3.3)	1.1 (0.3–3.9)	0.281
Cortisol, baseline (ng/ml)	174.2 ± 52.4	198.5 ± 47.5	0.123^a^
Cortisol, post-exercise (ng/ml)	268.5 ± 65.6	276.6 ± 64.2	0.694^a^
Δ Cortisol (ng/ml)	94.9 (54.3–130.9)	68.7 (45.1–97.2)	0.290
Testosterone, baseline (ng/ml)	4.5 ± 1.1	4.9 ± 1.1	0.269^a^
Testosterone, post-exercise (ng/ml)	3.7 (3.1–4.4)	4.7 (3.7–5.2)	0.049
Δ Testosterone (ng/ml)	− 0.6 (− 1.1 to − 0.2)	− 0.4 (− 0.8 to − 0.01)	0.292
Testosterone/cortisol, baseline	2.8 (2.1–3.1)	2.5 (2.1–3.3)	0.015
Testosterone/cortisol, post-exercise	1.5 (1.1–1.9)	1.5 (1.4–2.1)	0.116
Δ Testosterone/cortisol	− 0.5 (− 1.0 to − 0.3)	− 0.6 (− 1.6 to 0.1)	0.537
Myoglobin, baseline (ng/ml)	24.2 (15.5–32.9)	14.9 (10.1–22)	0.034
Myoglobin, post-exercise (ng/ml)	40.5 (28.0–99.3)	24.5 (19.2–32.9)	0.032
Δ Myoglobin (ng/ml)	18.6 (9.3–54.3)	10.3 (7.4–15.4)	0.052

*T1D* type 1 diabetes, *BMI* body mass index, *VO*_2_max maximal aerobic capacity, *LDH* lactate dehydrogenase, *TNF-α* tumour necrosis factor α

^a^Parametric test was used

The correlation analysis revealed that a higher level of HbA1c was associated with a lower myoglobin concentration at baseline (*r* = − 0.60, *P* = 0.014) and after exercise (*r* = − 0.71, *P* = 0.002). A longer duration of diabetes was correlated with a lower level of testosterone before (*r* = − 0.57, *P* = 0.022) and after exercise (*r* = − 0.58, *P* = 0.020), as well as with a lower level of LDH (*r* = − 0.51, *P* = 0.043). There was also a positive correlation between testosterone and vitamin D levels (*r* = 0.60, *P* = 0.015) and a tendency towards a negative correlation between vitamin D and zonulin concentrations before exercise (*r* = − 0.47, *P* = 0.06). In healthy subjects, only the correlation between vitamin D and testosterone levels was confirmed (*r* = 0.50, *P* = 0.007).

The estimated differences in outcomes between the groups (with adjustment for baseline values) after exercise are presented in Table 2. After adjustment, the groups differed in zonulin, LDH, and myoglobin levels, testosterone/cortisol ratio, and lactate concentration determined 20 min after exercise.

**Table 2:** Estimated difference after exercise between the T1D and matched control groups

Category	T1D group		Control group		Estimated difference (T1D–control group)	95% confidence interval	*p*-value
	Before exercise	After exercise	Before exercise	After exercise			
Zonulin (ng/ml)	13.6 ± 5.6	15.2 ± 7.1	10.5 ± 2.7	14.5 ± 5.1	− 3.0	− 5.5; − 0.6	0.017
LDH (U/l)	191.1 ± 65.9	278.8 ± 86.8	170.5 ± 63.1	184.5 ± 56.1	74.3^a^	42.7; 106.0	< 0.001
TNF-α (pg/ml)	3.3 ± 1.9	6.0 ± 3.0	3.0 ± 1.8	5.1 ± 2.4	0.7	− 0.8; 2.1	0.351
Cortisol (ng/ml)	174.2 ± 52.4	268.5 ± 65.6	193.9 ± 41.0	274.6 ± 54.9	13.4	− 18.2; 45.0	0.398
Testosterone (ng/ml)	4.5 ± 1.1	3.9 ± 1.2	4.9 ± 1.1	4.8 ± 1.5	− 0.6	− 1.4; 0.1	0.107
Myoglobin (ng/ml)	40.2 ± 60.3	74.6 ± 88	16.5 ± 10.0	27.8 ± 12.5	12.2	0.7; 23.8	0.039
Testosterone/cortisol indicator	2.8 ± 1.2	1.5 ± 0.6	2.7 ± 0.8	1.9 ± 0.8	− 0.4	− 0.8; − 0.1	0.020
Lactate, baseline vs. 3 min post-exercise (mmol/l)	1.6 ± 0.2	13.7 ± 3.4	1.6 ± 0.2	12.9 ± 2.9	0.8	− 1.1; 2.76	0.411
Lactate, baseline vs. 20 min post-exercise (mmol/l)	1.6 ± 0.2	7.9 ± 2.8	1.6 ± 0.2	6.2 ± 1.9	1.7	0.3; 3.0	0.081

*T1D* type 1 diabetes, *LDH* lactate dehydrogenase, *TNF*-α tumour necrosis factor α, *VO*_2_max maximal aerobic capacity

^a^With significant interaction included

After adding the VO_2_max variable to the model, group differences remained significant for zonulin (*P* = 0.018), LDH (*P* < 0.001), and testosterone/cortisol indicator (indicator of anabolic-catabolic balance) (*P* = 0.008), as well as borderline-significant for myoglobin (*P* = 0.058)

## Discussion

This was the first study to comprehensively compare muscle damage markers and zonulin concentration after a physical capacity test in men with T1D and matched healthy controls.

### Muscle damage

The main findings indicate that after a single short exercise of maximum intensity, the individuals with T1D compared with the control group (after adjustment for baseline values) presented significantly higher levels of myoglobin, LDH, and lactate determined 20 min after the test. No significant correlations were observed between vitamin D concentration and the levels of muscle damage markers after maximum exercise. The concentration of zonulin differed between the compared groups at baseline but no differences were observed after the exercise. When the differences after the exercise were adjusted for the baseline values, the zonulin level differed significantly between the groups.

The molecular and cellular mechanisms of skeletal muscle adaptation to exercise training are unclear. Owing to the mechanical, metabolic, and inflammatory processes that occur during and after physical activity, muscle cells become damaged [3]. In healthy individuals, the increase in metabolite levels associated with muscle damage has no consequences for health. However, in patients with T1D, at an increased risk of renal complications related to diabetes pathophysiology, excessive amounts of intramuscular proteins may be released into the bloodstream and precipitated in the renal tubules, negatively affecting renal function [22]. Damage to muscle cells resulting from physical activity initiates a series of immune reactions, including cytokine production and systemic leukocyte release [23]. Therefore, deep muscle damage and inflammatory responses observed with maximum-intensity exercise can influence glycaemic control in T1D subjects. LDH is the key enzyme that catalyses the interconversion of pyruvate and lactate, thus regulating the homeostasis of cellular pyruvate and lactate [24]. Higher LDH activity was shown to occur in healthy individuals after exercise because of changes in the permeability of cell membranes and cell necrosis [25]. The major role is attributed to increased cell membrane permeability due to changes in potassium, glucose, and albumin concentrations in the extracellular fluid, tissue hypoxia, and temporary modifications of the metabolic path [25]. These phenomena are typical in patients with T1D. Some authors suggest that an increase of LDH activity in serum occurs after prolonged exercise [25]. Numerous tests performed in smaller and more athletic groups of people, as well as in animals have revealed a higher increase in the activity of this enzyme in untrained groups [25]. Since the release of enzymes from cells is conditioned by energy supply, it can be assumed that working muscles deplete more quickly in less physically fit individuals [25]. In the presented study, VO_2_max was lower in T1D patients than in healthy controls. Studies by Fernando et al. [26] and Sandler et al. [27] revealed that the glycosylation of collagen fibres that occurs in diabetics, limiting joint mobility, can also arise in the lungs and heart. These changes may lead to decreased lung function test results. It can therefore be speculated that collagen fibre glycosylation in T1D patients may have contributed to the reduced VO_2_max levels that were observed in the present study. In addition, the decreased aerobic capacity in T1D may be due to reduced mitochondrial oxidative capacity [28]. The same results were reported in other papers [29, 30]. After adjusting the ANCOVA model for the VO_2_max variable, the differences between the groups remained significant (*P* < 0.001). These findings may confirm a higher permeability of cell membranes in people with T1D.

The study implied a higher lactate concentration 20 min after completion of maximum-intensity exercise in the T1D group compared with the control. The higher concentration of lactate may contribute to the accompanying hyperglycaemia. Lactate may act as a potential alternative substrate for glucose [31] and provide gluconeogenic precursors for hepatic glucose production [32]. Moreover, higher levels of lactate may sharply inhibit the insulin impact on peripheral glucose uptake, which is an action similar to that of counterregulatory hormones [33]. Peri-exercise lactate monitoring in T1D may provide additional information to optimize physical activity [34]. Myoglobin is a protein responsible for the storage of oxygen in striated muscle tissue. Only myoglobin released from damaged muscle cells, both of the heart and of skeletal muscles, enters the bloodstream and sometimes also the urine. The study showed that high-intensity exercise increased the level of myoglobin in healthy people [35]. On the basis of model simulations, the contribution of myoglobin oxygenation to total heme oxygenation can be significantly different under pathophysiological conditions, such as diabetes and peripheral arterial disorder [36]. In the present study, at baseline and after exercise, patients with T1D exhibited higher myoglobin values than the healthy controls. The higher concentration of myoglobin after maximum-intensity exercise may result from a reduced ability of T1D individuals to tolerate exercise and, at the same time, which is characteristic of this population, impaired exercise tolerance [37]. Furthermore, an inverse correlation between HbA1c and myoglobin level was observed in the T1D group. Although there was a difference between the groups, all results were in the normal range.

The analyses did not reveal differences in vitamin D concentration between the T1D subjects and the healthy controls. There was only a positive correlation between vitamin D and testosterone level in both groups and a borderline-negative correlation with zonulin concentration prior to exercise. The optimal level of 25(OH)D in blood plasma supports the muscular system during and after physical activity, helping maintain the correct concentrations of pro- and anti-inflammatory cytokines (mainly TNF-α) and therefore inhibiting inflammatory responses that arise [38]. Plasma deficiency of this vitamin can lead to impaired motor coordination and increases the risk of muscle damage [15]. Skeletal muscle histology showed an association between fast-twitch fibre atrophy and a suboptimal 25(OH)D level, which confirms the importance of vitamin D for normal muscle function [39]. In the literature, there are studies describing the effect of vitamin D supplementation on the level of muscle damage under the influence of exercise [19, 40]; however, there is no such research in patients with T1D. Furthermore, epidemiological data suggest a possible relationship between vitamin D deficiency and the worldwide occurrence of T1D [41, 42]. The presented results are in line with a recent meta-analysis which cannot conclude that vitamin D supplementation exerts an effect on post-exercise muscle recovery. Most likely, the anti-inflammatory action of vitamin D is faster than the recovery of tissue structure and function [43].

### Zonulin concentration

Nowadays, the role of the gut microbiota and physical activity is gaining more and more attention. Regular and moderate physical activity brings about many beneficial functional changes in gastrointestinal diseases; however, as the intensity of physical activity rises, the risk of intestinal microflora disorders increases [44].

Zonulin concentration is elevated in patients with T1D and in animal models of T1D, in both the prediabetic and diabetic stages [45–47]. Elevated zonulin levels are found in the plasma of 75% of patients with T1D [45]. The presented results confirm this finding. Men with T1D exhibited higher zonulin concentrations before and after exercise than controls (Table 1). Increased intestinal permeability, commonly referred to as ‘leaky gut,’ is associated with T1D and has been widely described in the literature [48]. Taking into account the presence of resting zonulin concentration in T1D and its level variation after physical activity, it seems advisable to conduct more studies examining patterns of zonulin concentration changes in T1D in response to physical activity of varying intensity. Feng et al. implied that an exercise program in adults resulted in a relative improvement in a biomarker of intestinal barrier integrity, indicating a potential mechanism by which longer-term exercise might improve this integrity [49]. This is another reason why the healthy control group with higher physical capacity exposed a lower zonulin level. It is believed that muscle unaccustomedness to high-intensity eccentric exercise, and not eccentric exercise per se, is the trigger for muscle damage, as reflected by muscle damage biomarkers [50]. One should emphasize the importance of optimal vitamin D concentration, which was shown in our study to be associated (with borderline significance) with the baseline level of zonulin. The link between diabetes development and intestinal permeability strongly suggests that increased intestinal permeability is a causal factor in T1D.

The finding of lower testosterone concentrations in T1D patients compared with the control group reported in this research is consistent with observations by other authors [51–56]. The decrease in testosterone levels in T1D men is explained by the presence of features of a complex of metabolic diseases [53, 54], iatrogenic hyperinsulinemia and insulin resistance [55]. On the other hand, some studies revealed higher testosterone concentrations in T1D individuals [52]. This may be caused by the fact that patients with T1D need more insulin to control glucose than healthy people with normal beta cells. Exogenous hyperinsulinemia in T1D patients can stimulate testosterone production and induce high serum testosterone levels.

The literature seems to be deficient in studies in which observation of changes in testosterone and cortisol levels would be applied to monitor fatigue and overtraining in T1D. Monitoring the anabolic-catabolic balance in T1D may contribute to a better understanding of recommendations regarding the intensity and duration of physical activity in this group of patients. In the presented research, significant changes were found in this indicator in T1D patients compared with the control group after a single effort of maximum intensity. Observation of changes in the testosterone/cortisol ratio is commonly used in the practice of sports physiology in healthy people [57, 58]. An increase in cortisol concentration and a decrease in testosterone concentration can further intensify the catabolic environment at the tissue level, thereby reducing muscle strength and overall performance [59].

The study revealed a correlation between serum vitamin D and testosterone level in both studied groups. A meta-analysis of 10 human randomized controlled trials provided evidence of an effect of vitamin D on total testosterone [60]. The same findings concerned males with type 2 diabetes [61]. To date, no studies on this issue have been conducted among patients with T1D.

Another finding was a higher lactate level 20 min after exercise in T1D individuals than in the healthy controls (Table 2). This was probably related to the lower physical capacity of the T1D group. In patients with T1D, the increase in blood lactate concentrations after physical exercise was shown to be larger in older subjects and did not depend on gender, insulin administration method, or training in a sports club vs. recreational play [62]. Compared with aerobic exercise, a cardiopulmonary exercise test was associated with higher lactate levels in adult men with T1D [34].

One may be concerned about a higher level of muscle damage markers and zonulin concentration in the context of renal function in patients with T1D. Recent studies have shown that even prolonged intense exercise resulting in elevated muscle damage and inflammatory biomarkers do not affect renal function [12]. However, a study on eccentric exercise in men with moderately controlled T1D indicated skeletal muscle alterations after damaging exercise, suggesting that longer recovery might be necessary after intense effort [63].

## Limitations

The study has some limitations. First, the diet was not analysed. In other studies, supplementation of protein was shown to influence exercise-induced muscle stress responses by changing cellular metabolism and inflammatory pathways. The supply of essential amino acids is critically related to muscle protein synthesis and can promote skeletal muscle hypertrophy in response to chronic resistance training [64]. Intake of branched chain amino acids favours post-exercise muscle recovery and may improve muscle function [65]. However, the evidence linking protein intake with a benefit in endurance sports has not been clearly established [64]. Other nutritional products, such as polyphenols, omega-3 acids, vitamin D, or vitamin C, could also play an important role in alleviating EIMD [4].

Muscle damage markers and inflammatory conditions can occur hours after exercise is over, which could constitute another limitation of the present investigation. In addition, the study only included male participants and patients using personal insulin pumps.

## Conclusions

Maximum-intensity exercise increased the levels of muscle damage markers and zonulin concentration in men with T1D. In patients with a lower level of physical activity, very-high-intensity exercise should be recommended with caution. Observation of changes in the anabolic-catabolic index may help individualize the intensity of physical activity undertaken by patients with T1D.

## Data Availability

Data are available from the corresponding author upon reasonable request.
